# Isolation and identification of major bacterial contaminants in beef and abattoir environments at Nekemte municipal abattoir, western Ethiopia: a cross-sectional study, 2024-25

**DOI:** 10.1186/s12866-026-05349-1

**Published:** 2026-06-30

**Authors:** Abdi Kidane Mengesha, Jobir Tesfa Adino, Tariku Desta Gelgelu

**Affiliations:** 1 Department of Pathology, School of Veterinary Medicine, Wollega University, Nekemte, Ethiopia; 2 Department of Microbiology, School of Veterinary Medicine, Dambi Dollo University, Dambi Dollo, Ethiopia; 3 Department of Biomedical Science, School of Veterinary Medicine, Borana University, Yaballo, Ethiopia

**Keywords:** Bacterial contamination, Meat hygiene, Beef, *Salmonella spp*, *Staphylococcus spp.*, *Escherichia coli*, Ethiopia

## Abstract

**Background:**

Foodborne disease caused by bacterial contamination of meat represent a major global public health challenge, particularly in developing countries where hygienic practices during meat processing are often inadequate.

**Objectives:**

This study aimed to isolate and identify major bacterial contaminants from beef, abattoir equipment and workers hands at Nekemte municipal abattoir, western Ethiopia.

**Methods:**

A cross sectional study was conducted from November 2024 to January 2025. A total of 92 samples were collected, including meat (*n* = 37), workers’ hand swabs (*n* = 30) and knife swabs (*n* = 25). Standard bacteriological culture techniques and biochemical tests were used for pathogen identification. Data were analyzed using SPSS version 16. Chi-square tests were performed to assess associations, with statistical significance set at *p* < 0.05.

**Results:**

The overall prevalence of bacterial contamination was 57% (52/92). Knife swabs showed the highest contamination rate (64%), followed by workers’ hand swabs (57%) and meat samples (51%). *Salmonella spp*. were the most frequently isolated pathogens (42%), followed by Presumptive *Staphylococcus spp.* (29%) and *Escherichia coli* (29%). No statistically significant difference in bacterial contamination was observed among the different sample types (*p* = 0.615).

**Conclusion:**

The high prevalence of bacterial contamination indicates inadequate hygienic practices in the abattoir. Strengthening sanitation measures, implementing structured training programs and adopting standardized food safety systems are recommended to improve meat safety.

## Introduction

Foodborne diseases remain a major global public health concern. According to the World Health Organization (WHO), approximately 600 million cases of foodborne illness and 420,000 deaths occur annually due to the consumption of contaminated food [1]. Among bacterial pathogens, *Salmonella* species are responsible for an estimated 98.8 million cases of gastroenteritis and approximately 155,000 deaths worldwide each year [2]. In the United States of America alone, the Centers for Disease Control and Prevention (CDC) reports approximately 1.35 million infections, 26,500 hospitalizations and 420 deaths annually due to *Salmonella* infection [3].

Meat is an important source of high- quality protein and essential nutrients in the human diet. However, it is highly susceptible to microbial contamination due to its rich nutrient composition and moisture content (70–73%water, 20–22% protein and approximately 5% lipids). This composition provides an ideal environment for microbial growth. Although muscle tissue from healthy animals is sterile, contamination frequently occurs during slaughtering, dressing, processing, transportation and marketing [4].

Microbial contamination may originate from animals skin, gastrointestinal tract, processing equipment, water sources, handlers hands, air processing equipment, water sources, hygiene practices during meat handling significantly increase the risk of contamination with pathogenic bacteria such as *Escherichia coli*, *Salmonella spp*. and Presumptive *Staphylococcus spp.* [5].

Foodborne pathogens are responsible for more than 200 diseases, ranging from mild gastrointestinal disorders to severe systemic infections. Children under five years of age account for approximately 40% of foodborne disease cases globally [6]. The economic burden associated with foodborne diseases includes healthcare costs, productivity loss and trade restrictions. Several bacterial pathogens are commonly associated with meat contamination, including *E. coli*, non-typhoid *Salmonella*, *Campylobacter*,* Listeria monocytogenes*,* Presumptive Staphylococcus spp.* and *Clostridium* species [7]. These pathogens may originate from infected animals, contaminated equipment, handles or unsanitary environmental conditions.

In Ethiopia, limited data are available regarding bacterial contamination along the meat production chain, particularly at municipal abattoirs. Therefore assessing bacterial contamination and identifying major pathogens is essential for developing effective intervention strategies.

### The objectives of the study

- ➢ To determine the prevalence of bacterial contamination in beef, workers’ hand swabs and knife swabs at Nekemte Municipal Abattoir.
- ➢ To isolate and identify major bacterial contaminants associated with meat and the abattoir environment.
- ➢ To assess differences in contamination levels among sample types.

## Materials and methods

### Study area

The study was conducted at Nekemte Municipal Abattoir, located in Nekemte Town, East Wollega Zone, Oromia Regional State, Western Ethiopia and approximately 335 Km west of Addis Ababa. The town lies at an altitude ranging from 1960 to 2170 m above sea level and receives an annual rainfall of 1500–2200 mm. The abattoir slaughters an average of 30 cattle per day.

Nekemte Municipal Abattoir was purposively selected because it is the main slaughter facility serving Nekemte town and surrounding districts and represents routine slaughterhouse practices common to municipal abattoirs in western Ethiopia.

At time of the study, the abattoir had limited hand washing facilities, inconsistent water supply, and no clearly separated clean and dirty processing areas. Knifes and equipment were commonly washed without disinfectant, and no refrigeration facilities were available for storage.

### Study design

A cross-sectional study was conducted from November 2024 to January 2025 to isolate and identify major bacterial contaminants from beef and abattoir environments. The study was conducted during a limited three-month period and therefore reflects contamination levels during the study season rather than year-round trends.

### Sample size and sampling techniques

A total of 92 samples were collected using purposive sampling over the entire study period (November 2024 to January 2025). Sampling was conducted two to three times per week, with approximately 6 to 8 samples collected per sampling day based on the daily slaughter rate of approximately 30 cattle and operational feasibility during the study period. The sample size was considered adequate to provide a representative snapshot of contamination levels within the abattoir during the study period.

Although a formal sample size calculation was not performed before data collection, a post-hoc justification was conducted using an expected contamination prevalence of 50%, 95% confidence level, and operational feasibility within the study period. The sample size of 92 provided an acceptable preliminary estimate of contamination prevalence in the abattoir environment. However, the relatively small sample size may have reduced the statistical power to detect significant differences among sample types, and this should be considered as a limitation of the study. So 37 meat sample, 30 workers hand swabs and 25 knife swabs samples were collected aseptically.

### Sample collection and transportation

Approximately 25 g of beef samples were collected from the surface of carcasses immediately after slaughter and placed into sterile container for bacteriological analysis.

Hand swab samples were collected from workers during active slaughter operations, after they had handled carcasses. Knife swabs were collected during processing, immediately after use and before cleaning or sterilization.

Swab samples were collected using sterile cotton swabs moistened with buffered peptone water. A 100 cm^2^ surface area was swabbed according to ISO 17,604 (2005) guidelines. Swabs were placed into sterile tubes containing 10 ml of buffered peptone water and transported in an ice box to the microbiology laboratory for analysis.

### Bacteriological isolation and identification

#### Isolation and Identification of presumptive *Staphylococcus spp*.

Isolation and identification of presumptive *Staphylococcus spp.* were conducted following standard microbiological procedure based on ISO 6888-1:1999 guidelines. Samples were pre-enriched in buffered peptone water and a loopful (50ul) of the enriched culture was streaked onto tryptic soya agar supplement with 5% sheep blood and incubated aerobically at 37 °C for 24 h.

Presumptive colonies were subcultured onto mannitol Salt Agar (MSA). Yellow colonies surrounded by yellow zones after incubation at 37 °C for 24 h were considered presumptive *S. aureus* based on mannitol fermentation. Further identification was performed based on, colony morphology (round, smooth, golden-yellow colonies), hemolytic pattern on blood agar, Gram staining (Gram-positive cocci in cluster) and catalase test (positive reaction indicated by bubble formation were observed. However, isolates were not confirmed using the coagulase test; therefore, the isolate were reported as presumptive *Staphylococcus spp.*

#### Isolation and identification of *Escherichia coli*

Detection of *Escherichia. coli* was performed following ISO 16649-2:2001 guidelines. Swab sample were inoculated into 9 ml of modified Tryptone Soya Broth and incubated at 41 °C for 24 h. Following enrichment, sample were streaked onto MacConkey agar and incubated at 37 °C for 24 h.

Pink lactose fermenting colonies were considered presumptive *Escherichia coli*. These colonies were further subcultured on to Eosin Methylene Blue (EMB) Agar. Colonies exhibiting a characteristic Metallic green sheen were identified as presumptive *Escherichia coli*. Further identification included Gram staining (Gram negative rods) and biochemical characterization: oxidase negative, indole positive, methyl red positive, Voges-Proskauer negative, and TSI reaction showing alkaline slant/acid butt with no hydrogen sulfide (H₂S) production.

#### Identification and Isolation of *Salmonella spp*.

For *Salmonella* isolation, 10 g of each sample was pre-enriched in lactose broth and incubated at 37 °C for 24 h. Selective enrichment was performed using selenite Broth, incubated at 37 °C for 24 h [8]. After enrichment, samples were streaked onto Bismuth Sulfite Agar (BSA) and incubated at 37 °C for 24 h [9].

Presumptive *Salmonella* colonies were identified based on; black or greenish colonies on BSA, Gram negative rod by gram stain and also identified by secondary biochemical test and characterized by oxidase negative by oxidase test, indole test negative, methyl red test positive, voges-proskauer (VP) test negative and TSI test positive with alkaline slant/acid butt with H_2_S production. Molecular or serological confirmation was not performed.

### Data analysis

Data were entered into Microsoft Excel and analyzed using statistical package for social science (SPSS) version 16. Descriptive statistics were used to calculate: prevalence (%) and frequency distributions.

The chi-square (X^2^) test was used to assess association between sample types and bacterial contamination. Statistical significance was determined at a 95% confidence interval with p-value < 0.05 considered statistically significant.

## Results

### Overall prevalence of bacterial contamination

Out of 92 total samples examined, 52 (57%) were positive for bacterial contamination.

### Contamination by sample type

Table 1 shows the highest contamination rate was observed in knife swabs (64%), followed by workers hand swabs (57%) and meat samples (51%). The chi-square analysis showed no statistically significant association between sample types (meat, hand swabs, and knife swabs) and bacterial contamination (X^2^ = 0.972, *P* = 0.615). However, the relatively small sample size may limited the statistical power to detect significant differences.

**Table 1:** Prevalence of bacterial contamination by sample type

Sample type	Positive (*n*)	Negative (*n*)	Prevalence (%)	95% Cl
Meat	19/37	18	51%	35.5–67.0
Worker hand swab	17/30	13	57%	37.4–74.5
Knife swab	16/25	9	64%	42.5–82.0
Total	52/92	40	57%	45.8–66.7

### Distribution of bacterial isolates

Table 2 shows among the 52 bacterial isolates identified, *Salmonella spp*. were the most frequently isolated pathogens, accounting for 42% (22/52) of all isolates. Most *Salmonella spp.* isolates were recovered from knife swabs (16 isolates), while the remaining isolates were obtained from workers’ hand swabs. Presumptive *Staphylococcus spp. and Escherichia coli* each accounted for 28.8% (15/52) of the total isolates.

**Table 2:** Frequency and percentage of isolated bacterial pathogens

Bacterial Isolate	Meat (*n*)	Hand swab (*n*)	Knife Swab (*n*)	Total *n* (%)
*Salmonella spp.*	0	6	16	22(42%)
*Presumptive Staphylococcus spp.*	10	3	2	15(29%)
*Escherichia coli*	4	11	0	15(29%)
Total	19	17	16	52(100%)

Presumptive *Staphylococcus spp.* were predominantly isolated from meat samples, whereas *Escherichia coli* was more frequently isolated from workers’ hand swabs. Only one predominant bacterial isolate was recorded from each positive sample based on the primary culture characteristics and selective isolation procedure employed in this study. Mixed bacterial growths were not further characterized; therefore, co-occurrence of multiple bacterial pathogens within the same sample was not observed.

## Discussion

The present study revealed an overall bacterial contamination prevalence of 57% among sample collected from beef, workers hand and knifes at Nekemte municipal abattoir. This finding indicates a substantial level of microbial contamination within the meat production environment and highlights potential public health risk associated with beef consumption in the study is compared to findings reported in similar investigations conducted in developing countries, where inadequate hygienic practices and limited sanitation infrastructure contributed significantly to meat contamination. Studies conducted in Nigeria and other African regions have reported similar contamination levels in abattoir environments, emphasizing the persistent challenges of meat hygiene in resource limited setting [10, 11].

The highest contamination rate was recorded in knife swabs two third of the sample, followed by workers hand swabs (57%) and meat samples (51%). Although the chi-square test showed no significant association between sample type and contamination (*p* > 0.05). The higher contamination observed on knifes suggests inadequate sanitation and improper cleaning protocols. Knifes serve as direct contact surfaces during carcass processing, if not properly sterilized between slaughtering procedures, they can act as mechanical vectors, transferring pathogens from one carcass to another. This cross contamination pathways in well documented in meat hygiene literature.

Similarly, contamination of workers’ hands indicates poor personal hygiene practices. Handling meat without proper handwashing, using disinfectants or wearing protective gloves increases the risk of bacterial transmission. In additional, lack of regular training and awareness among workers regarding food safety practices may contribute to adequate hygiene behavior. Addressing both infrastructure and capacity building is essential for sustainable improvement. The findings support previous reports indicating that food handlers play a critical role in microbial dissemination within meat processing environments [12].

*Salmonella spp*. were the most frequently isolated pathogens (42%). However, no statistically significant association was observed between sample type and bacterial contamination.

*Salmonella* is commonly associated with the gastrointestinal tract of animals and can contaminate carcass during slaughtering if proper handing is not maintained. The predominance of *Salmonella* in this study aligns with global reports identifying it as one of the leading causes of foodborne gastroentestitis. Its presence in meat and contact surfaces is a strong indicator of inadequate hygienic measures during slaughtering and carcass dressing.

The isolation presumptive *Staphylococcus spp.* (29%) suggests contamination originating from human (handlers), as this organism is commonly found on human skin, nasal passages and hands. Poor personal hygiene practices such as sneezing, coughing or handling meat without gloves can introduce this pathogen into the processing environment. The presence of Presumptive *Staphylococcus spp.* is particularly concerning due to its ability to produce heat stable enterotoxins, which may cause food poisoning even after cooking.

The detection of *Escherichia coli* (29%) indicates fecal contamination and inadequate sanitary conditions. *E. coli* is widely recognized as an indicator organism for hygiene quality in meat production. Its presence suggests improper slaughtering techniques, contamination from intestinal contents or unsanitary water used during processing. Comparable findings have been reported in studies assessing microbial contamination in abattoirs and butcher shops, where *E. coli* was frequently isolated from meat and contact surfaces [10].

The detection of *Salmonella spp*., Presumptive *Staphylococcus spp*., and *Escherichia coli* across different sample types suggests the existence of multiple contamination routes, including fecal contamination and cross-contamination from equipment and handlers. However, co-occurrence of multiple bacterial pathogens within the same sample was not assessed in this study because only one predominant bacterial isolate was recorded from each positive sample. These findings highlight significant public health concerns, particularly in areas where raw or uncooked meat consumption is common. The risk is further exacerbated by inadequate refrigeration and transportation systems, which allow bacterial proliferation.

Although no statistically significant association was observed between sample type and contamination (*p* = 0.615), this may be attributed to the relatively small sample size, which can reduce statistical power. The observed numerical differences (64% vs. 51%) suggest potential practical significance that may not have been detected statistically. Future studies with larger sample sizes are recommended to improve inferential strength.

Foodborne infections not only affect individual health but also impose economic burdens through healthcare costs and productivity losses. Therefore, improving hygienic practices within abattoir is essential for safeguarding community health. Although this study has some limitations that should be recognized; despite this the results provide good insights into the level of bacterial contamination at Nekemte Municipal Abattoir. Purposive (non-probability) sampling might have created selection bias and reduced the diversity of samples obtained. The results, therefore, do not necessarily apply to all slaughter operations and other abattoirs and caution should be exercised with regard to generalizations. Furthermore, due to the small number of cases and only one municipal abattoir, there might be less statistical power in this study and the findings may not apply to other sites.

- The study relied solely on conventional culture and biochemical identification methods without molecular confirmation.
- Antimicrobial susceptibility testing was not performed.
- The sample size was relatively limited to one municipal abattoir.
- Future studies incorporating molecular techniques and antimicrobial resistance profiling would provide more comprehensive epidemiological data.

## Conclusion

This study demonstrated a high prevalence of bacterial contamination in beef and abattoir contact surfaces at Nekemte Municipal Abattoir. The major bacterial isolates identified were *Salmonella spp.*, presumptive *Staphylococcus spp*., and *Escherichia coli*, indicating inadequate hygienic and sanitation practices during meat processing. The findings highlight the potential public health risks associated with contaminated meat and food safety control measures within the abattoir. Future studies incorporating molecular confirmation and antimicrobial susceptibility testing are recommended.

### Recommendations

Based on the findings of this study, the following recommendations are proposed:

- Regular cleaning and sterilization of knifes and processing equipment should be enforced using hot water at 82 °C or 0.5% chlorinated disinfectant solution between carcasses.
- Abattoir workers should receive hygiene and food safety training at least once every three months, with pre and post training performance assessments.
- Continuous water availability per slaughter day should be maintained to support proper cleaning and sanitation activities.
- Separate clean and dirty processing areas should be established to minimize cross-contamination during slaughter operations.
- Routine microbiological monitoring of meat, equipment, and workers hands should be implemented as part of the abattoir food safety program.
- Future studies should include molecular identification techniques and antimicrobial susceptibility testing for better characterization of bacterial isolates.

## Data Availability

The datasets generated and analyzed during the current study are available from corresponding author upon reasonable request.

## Abbreviations

BPW: Buffered Peptone Water
BSA: Bismuth Sulfite Agar
CDC: Centers for Disease Control and Prevention
EMB: Eosin Methylene Blue
ISO: International Organization for Standardization
LB: Lactose Broth
MBD: Meat-borne Disease
MR: Methyl Red
MSA: Mannitol Salt Agar
SB: Selenite Broth
SPSS: Statistical Package for the Social Sciences
SVM: School of Veterinary Medicine
TSI: Triple Sugar Iron Agar
TSA: Tryptic Soy Agar
VP: Voges–Proskauer
WHO: World Health Organization
